# AN UNCONTROLLED OPEN PILOT STUDY TO ASSESS THE ROLE OF DIETARY ELIMINATIONS IN REDUCING THE SEVERITY OF ATOPIC DERMATITIS IN INFANTS AND CHILDREN

**DOI:** 10.4103/0019-5154.53187

**Published:** 2009

**Authors:** Sandipan Dhar, Rajib Malakar, Raghubir Banerjee, Saswati Chakraborty, Jayanti Chakraborty, Susmita Mukherjee

**Affiliations:** *From the Department of Pediatric Dermatology Kolkata, India*; 1 *From the Department of Pediatric Medicine Kolkata, India*; 2 *From the Department of Dietetics, Institute of Child Health, Kolkata, India*

**Keywords:** *Atopic dermatitis*, *dietary elimination*, *severity score*

## Abstract

**Background:**

The severity of atopic dermatitis (AD) has been reported to be reduced by dietary eliminations in a subset of patients with AD.

**Aims:**

To assess the reduction of the severity of atopic dermatitis in infants and children after eliminations of certain dietary items.

**Materials and Methods:**

The study group comprised of 100 children with atopic dermatitis. Their severity of itching, surface area of involvement, and SCORAD index were measured. Patients who did not have any systemic disease or were not on systemic corticosteroids were included in the study. Selected patients were advised to strictly adhere to a diet excluding milk and milk products, all kinds of nuts and nut-containing foods, egg and egg-containing foods, seafish and prawns, brinjal and soyabean for a period of 3 weeks. Instead of these avoided items, the food items to be included freely to maintain proper nutrition were dal and dal products, rohu fish, chicken, and fruits. All the preintervention parameters were measured again after 3 weeks.

**Results:**

There was a statistically significant reduction in severity scores after dietary elimination alone.

**Conclusion:**

Dietary elimination helped to alleviate symptoms and signs in a subset of infants and children with AD.

## Introduction

Atopic dermatitis is an itchy chronic or chronically relapsing inflammatory dermatosis that is characterized clinically by itchy papules that become excoriated and lichenified and typically have a flexural distribution. It is generally associated with other atopic conditions like bronchial asthma and allergic rhinitis in the individual or other family members.[1–4]

Numerous aggravating factors and possible etiological agents have been proposed as triggers for atopic dermatitis. The possible role of dietary factors in this regard has been emphasized by recent controlled studies. From a qualitative point of view, diet can be adapted to eliminate foods that are thought or are proven to play a pathogenic role. Dietary elimination is suggested for patients with atopic dermatitis either for diagnostic reasons to establish the presence of food allergies, for therapy, or as a preventive measure in the newborn at risk.

In an open-pilot study, we investigated the feasibility of dietary eliminations in the Indian scenario and also assessed the effect it has on Indian children with atopic dermatitis.

## Materials and Methods

A total of 100 children attending the pediatric dermatology outpatient department of the Institute who had atopic dermatitis and were between the ages of 6 months to 12 years were candidates for inclusion in the study. They were not included in the study if one had a history of other systemic disease or was on systemic corticosteroids.

Selected patients were advised to strictly adhere to a diet excluding the following items for a span of 3 weeks:

1. Milk and milk products
2. All kinds of nuts and nut-containing foods
3. Egg and egg-containing foods
4. Seafish and prawns
5. Brinjal and soyabean

Instead of these avoided items the following foods were included freely to maintain proper nutrition

1. Dal and dal products
2. Rohu fish
3. Chicken
4. Fruits

Infants who were 6 months to 12 months old were given protein hydrolysate formula (SimylMCT) instead of milk.

In the current study, we tested the hypothesis that specific dietary eliminations alone, in patients with atopic dermatitis, can lead to significant reduction in signs and symptoms of the disease.

The primary endpoint with respect to efficacy of the dietary eliminations assessed after 3 weeks was the presence of significant alteration in disease activity as measured by the SCORAD index, assessment of the surface area of involvement (Wallace Rule of Nine), and the degree of pruritus. SCORAD index (a scoring system to measure the severity of atopic dermatitis designed by the European Task Force on atopic dermatitis)[5] has the following signs: (1) vesiculation, (2) excoriation, (3) crusting, and (4) edema. Each sign has four scoring points from 0 to 3. Pruritus was also divided into four scoring points depending on the grade or severity of the symptom.

All data analysis was carried out according to a pre-established analysis plan. t distribution statistics were applied to obtain paired observations on the pre and post intervention population and to assess the presence of significant differences between the means of the two sets of observations.

## Results

Eligible participants were recruited from November 2002 to October 2003. Follow-up assessment of each patient was done 3 weeks after his/her inclusion in the study. Baseline demography and pre-intervention clinical characteristics of the study group have been highlighted in Table 1. The male to female ratio of the study group was 0.92. The clinical characteristics of the study group at follow-up after 3 weeks of dietary eliminations have been given in Table 2.

**Table 1:** Baseline demography and preintervention characteristics of atopic children

Characteristic (n = 100)	Mean	Standard deviation	Range	Minimum	Maximum
Age in months	48.32	34.34	138	6	144
Severity of itching	1.89	0.65	2	1	3
Surface area of involvement	15.32	8.14	37	3	40
SCORAD index	7.22	2.49	10	2	12

**Table 2:** Clinical characteristics of the study group at follow-up after 3 weeks of dietary eliminations

Characteristics (n = 100)	Mean	Standard deviation	Range	Minimum	Maximum
Severity of itching	0.64	0.959	3	0	3
Surface area	3.760	6.142	25	0	25
SCORAD	2.272	2.083	7	0	7

When the disease characteristics parameters at follow-up were compared with that of the baseline using t distribution, a statistically significant difference was observed to have occurred in each of the parameters following 3 weeks of dietary eliminations.

A statistical comparison summary of the two datasets have been highlighted in Table 3.

**Table 3:** A statistical comparison summary of the two datasets obtained

Characteristics	Difference of means	Standard error of difference	t value	Probability
Severity of itching	1.25	0.12	10.79	<0.001
Surface area	11.56	1.02	11.34	<0.001
SCORAD	4.95	0.32	15.25	<0.001

## Discussion

In 100 patients diagnosed with atopic dermatitis by following specific dietary eliminations for 3 weeks, we found statistically significant lowering of all the recorded parameters of disease activity.

Children with atopic dermatitis tend to have a higher prevalence of food allergies; about 35% of children with moderate to severe atopic dermatitis have IgE mediated food allergies.[5,6] Giullet, *et al.* had found a direct correlation between the increased severity of atopic dermatitis and the presence of food allergies after evaluating 250 children with atopic dermatitis.[7] We hypothesize that food allergens may act as one of triggers in inducing and maintaining the clinical manifestations of atopic dermatitis and specific dietary eliminations in specific cases can significantly reduce the disease activity.

The value of dietary exclusion was studied in a group of 62 children with atopic dermatitis, all of whom had raised specific IgE to eggs. Specific egg exclusion from their diet resulted in an improvement of severity of the disease.[8] Atherton, *et al.* reported that two-thirds of the children with atopic dermatitis showed a marked improvement during a double-blind crossover trial of egg and milk exclusion. [9] In a prospective follow-up study of 34 patients with atopic dermatitis, 17 children with food allergy who were appropriately diagnosed with the use of double-blind, placebo-controlled food challenges and placed on an appropriate allergen elimination diet experienced a marked and significant improvement in comparison with 12 subjects who did not have food allergy and 5 children with food allergy who did not follow dietary elimination.[10]

By its design, the present study is an open and uncontrolled one and other triggers had not been taken into consideration. The study serves the purpose of being a qualitative pointer to future research in this direction.

In the early part of the century, Schloss[11] reported several cases of patients who had improvement in their eczematous skin lesion after avoiding specific foods. That report was followed by many others with conflicting findings and led to the controversy about the role of specific food allergens in atopic dermatitis. Until now, very little evidence-based literature can be found to address this issue. Formulation of the proper methods and indications of using specific dietary eliminations and their substitution by appropriate calorie containing diet in atopic dermatitis can help dermatologists to control the severity of atopic dermatitis in a subset of infants and children with a definite positive outcome.

## References

[1] Ohman S, Johansson SG (1974). Immunoglobins in atopic dermatitis, with special reference to IgE. Acta Derm Venereol (Stockh).

[2] Ohman S, Johansson SGO (1974). Allergen – specific IgE in atopic dermatitis. Acta Derm Venereol (Stockh).

[3] Jones HE, Inouye JC, McGerity JL, Lewis CW (1975). Atopic disease and serum immunoglobin E. Br J Dermatol.

[4] Uehara M (1989). Family background of respiratory atopy: A factor of serum IgE elevation in atopic dermatitis. Acta Derm Venereol Suppl (Stockh).

[5] Solley GO, Gleich GJ, Jordan RE, Schroeter AL (1976). Late phase of the immediate wheal and flare skin reactions: Its dependence on IgE antibodies. J Clin Invest.

[6] Eigenmann PA, Sicherer SH, Borkowski TA, Cohen BD, Sampson HA (1998). Prevalence of IgE – mediated food allergy among children with atopic dermatitis. Pediatrics.

[7] Guillet G, Guillet MH (1992). Natural history of sensitizations in atopic dermatitis. Arch Dermatol.

[8] Lever R, MacDonald C, Waugh P, Aitchison T (1998). Randomised controlled trial of advice on an egg exclusion diet in young children with atopic eczema and sensitivity to eggs. Pediatr Allergy Immunol.

[9] Atherton DJ, Soothill JF, Sewell M, Wells RS, Chilvers CE (1978). A double blind controlled crossover trial of an antigen avoidance diet I atopic eczema. Lancet.

[10] Sampson HA, Jolie PL (1984). Increased plasma histamine concentrations after food challenges in children with atopic dermatitis. N Engl J Med.

[11] Schloss OM (1915). Allergy to common foods. Trans Am Pediatr Soc.

